# An Unusual Presentation of Catatonia in Non-alcoholic Wernicke Encephalopathy

**DOI:** 10.7759/cureus.12537

**Published:** 2021-01-06

**Authors:** Saeed Ahmed, Tayo V Akadiri, Subhan Ata, Shahana Ayub

**Affiliations:** 1 Psychiatry and Behavioral Sciences, Nassau University Medical Center, East Meadow, USA; 2 Behavioral Health Sciences, Boston University School of Medicine, Boston, USA; 3 Anaesthesiology, Ocala Regional Medical Center, Ocala, USA; 4 Internal Medicine, BronxCare Health System, New York, USA; 5 Psychiatry, Cornerstone Healthcare System, Newburgh, USA

**Keywords:** wernicke encephalopathy, malnutrition, gait ataxia, neuropsychiatric syndrome, thiamine deficiency, catatonia, non-alcoholic wernicke's encephalopathy, korsakoff’s syndrome, wernicke-korsakoff syndrome, cerebellar dysfunction

## Abstract

Wernicke encephalopathy (WE) is an acute reaction to thiamine deficiency, which presents with the classic triad of ocular findings, cerebellar dysfunction, and confusion. However, thiamine deficiency can also present with several neuropsychiatric signs and symptoms other than the classical triad. We report a patient who presented with catatonia as a presenting feature of WE. The objective of this report is to recognize the presentation of catatonia in WE. Some cases of WE are missed by physicians; therefore, a high index of suspicion and appropriate investigations depending on presentation and clinical condition can result in prompt diagnosis and early management.

## Introduction

Wernicke encephalopathy (WE) is an acute neuropsychiatric syndrome resulting from thiamine (vitamin B1) deficiency, characterized by a triad of ophthalmoplegia, ataxia, and altered mental status (AMS). Thiamine deficiency is common in people with chronic alcoholism and can also be seen in people with severe malnutrition, prolonged parenteral nutrition, liver disease, hyperemesis gravidarum, malignancies, immunodeficiency syndromes, hyperthyroidism, severe anorexia nervosa, and after weight-loss (bariatric) surgery. This is a life-threatening illness and associated with significant morbidity and mortality. Unfortunately, WE is still underdiagnosed in clinical settings because it may not always show up with a classical presentation, which is present in only approximately 10%-16% of patients [1]. WE is reversible if detected and treated promptly. Without treatment, it can be disabling and life-threatening. Approximately 80% of patients with untreated WE precedes Korsakoff syndrome [2], which is characterized by amnesia, tremor, disorientation confabulation, and coma. At this stage, reversing the brain damage becomes difficult, and only a few individuals recover. The index case illustrates a rare presentation of WE; it will serve to inform clinicians about the clinical complexity of such cases.

## Case presentation

A 41-year-old African American male with a past medical history of hypertension, no known psychiatric illness, or history of substance abuse was brought to the emergency department with AMS. His mother called the emergency medical services (EMS), who found him in the basement. The mother reported that the patient had not eaten for almost 9-10 days prior. EMS found him to be lethargic, mumbling incoherent words, with urine in bottles around him. He was admitted to the medicine floor for AMS. On evaluation by the Psychiatry Consultation-Liaison (CL) team, the patient was disheveled, malodorous, and cachectic with a refusal to eat. He appeared confused, disoriented, and unresponsive to verbal or manual redirection and prompting. The patient had increased tone of his upper limbs and displayed waxy flexibility, posturing, and stereotypical repetition of meaningless phrases, e.g., "a man a woman that’s it". He stared blankly and had intermittent jerky movements of his left eye suggestive of nystagmus. Laboratory testing was remarkable for lactic acidosis (blood lactate concentration (3mmol/L)) and normal electrolyte levels. Cobalamin, folic acid level, thyroid function, HIV, Lyme, rapid plasma reagin (RPR), ammonia, liver enzymes, urine analysis, urine toxicology, blood culture, cerebrospinal fluid (CSF) culture, dementia, and autoimmune workup were all unremarkable. The CT scan of the brain and MRI brain were done in order to rule out acute intra-cranial events or abnormalities. The CT scans of the head, chest, abdomen, and pelvis were all unremarkable. Psychiatry CL team recommended lorazepam 2mg every eight hours for suspected catatonia. The patient continued to be confused with difficulty in communicating and responding to instructions and questions. On admission day four, the patient displayed ataxia and ophthalmoplegia. Neurological consultation excluded cerebrovascular accident and seizure disorder. WE was considered in the differential diagnosis, and thiamine 500mg IV thrice a day was administered for the next three days. On admission day seven, movement of the extremities and eating improved. The patient stated his name but continued to have staring spells and memory impairment with a Montreal Cognitive Assessment score of 22/30. An MRI revealed mild-to-moderate brain volume loss, with mild periventricular and subcortical T2 hyperintensities. By the second week of admission, lorazepam was tapered to 1mg twice a day. On admission day 12, the patient improved significantly, stating that his "mood was fine" and described his memory as "on and off". He was able to name objects, interact verbally, and his tone normalized. On admission day 14, the patient’s catatonia and mental status improved significantly. However, he still had nystagmus and ataxia. He was discharged to his mother’s home with aftercare appointments with the primary care physician, psychiatrist, neurologist, and ophthalmologist.

## Discussion

This case report highlights the diagnostic and treatment challenges associated with new-onset catatonia in a patient with WE. WE has been classically associated with alcoholism and malnutrition. It is an underdiagnosed condition, particularly in patients who have no prior history of alcohol use, as in this case report. We suspect that this patient developed WE due to malnutrition, as opposed to chronic alcohol use, which is much more common. WE is a clinical diagnosis where blood levels of thiamine and red blood cell transketolase activity can be low but are not very specific [3]. Physicians need to be vigilant and have a high index of suspicion for an atypical presentation that can be a complication of WE, such as psychosis [4,5] or catatonia, as in this case report. The prevalence of WE is high; however, presenting with the classic triad is less common, and so it is of utmost importance for healthcare providers to increase their awareness of other possible early presentations as previously suggested in the literature [6-8]. Patients with WE may not present with classical triad manifestations, particularly in the early stages, as in this case. Prompt diagnosis is essential as delay can lead to irreversible complications. There is a case report published previously by Braune et al., where a 28-year-old woman presented with catatonia along with classical symptoms of WE such as extraocular motility to lateral gaze and vertical nystagmus [9]. It suggests that WE is increasingly being recognized as a potential etiology of catatonia [10]. Little to no research has been proposed for treating catatonia in the context of WE; however, as exhibited in our case, early initiation of treatment of catatonia has potential benefits. Elucidation of the underlying WE and subsequent initiation of thiamine supplementation demonstrated a marked improvement in overall mentation. The current case emphasizes the importance of correcting medical causes while concomitantly treating catatonia with proven therapies. This case also highlights the importance of improving the early diagnosis and proper treatment of WE as we often come across complex patients and low healthcare provider awareness.

## Conclusions

This case highlights the importance of considering atypical presentations of WE. A complete medical workup and evaluation are important in individuals who present with catatonia in order to exclude medical causes. This case report also suggests that there is a plausible causal relationship between thiamine deficiency and catatonia, evidenced by the positive response to thiamine replacement.
